# Retreatment with brentuximab vedotin in patients with CD30-positive hematologic malignancies

**DOI:** 10.1186/1756-8722-7-24

**Published:** 2014-03-19

**Authors:** Nancy L Bartlett, Robert Chen, Michelle A Fanale, Pauline Brice, Ajay Gopal, Scott E Smith, Ranjana Advani, Jeffrey V Matous, Radhakrishnan Ramchandren, Joseph D Rosenblatt, Dirk Huebner, Pamela Levine, Laurie Grove, Andres Forero-Torres

**Affiliations:** 1 Washington University Medical School, St Louis, MO, USA; 2 City of Hope, Duarte, CA, USA; 3 University of Texas MD Anderson Cancer Center, Houston, TX, USA; 4 Hospital Saint-Louis, Paris, France; 5 University of Washington/Fred Hutchinson Cancer Center, Seattle, WA, USA; 6 Loyola University Medical Center, Maywood, IL, USA; 7 Stanford University Medical Center, Stanford, CA, USA; 8 Colorado Blood Cancer Institute, Denver, CO, USA; 9 Karmanos Cancer Institute, Detroit, MI, USA; 10 University of Miami Miller School of Medicine, Sylvester Comprehensive Cancer Center, Miami, FL, USA; 11 Takeda Pharmaceuticals International Company, Cambridge, MA, USA; 12 Seattle Genetics, Inc, Bothell, WA, USA; 13 University of Alabama at Birmingham, Comprehensive Cancer Center, Birmingham, AL, USA

**Keywords:** Hodgkin lymphoma, Systemic anaplastic large cell lymphoma, Brentuximab vedotin, Retreatment, Relapse

## Abstract

**Background:**

Brentuximab vedotin is a CD30-directed antibody-drug conjugate. Retreatment with brentuximab vedotin monotherapy was investigated in patients with CD30-positive Hodgkin lymphoma (HL) or systemic anaplastic large cell lymphoma (ALCL) who relapsed after achieving complete or partial remission (CR or PR) with initial brentuximab vedotin therapy in a previous study (ClinicalTrials.gov NCT00947856).

**Methods:**

Twenty-one patients with HL and 8 patients with systemic ALCL were retreated; 3 patients with systemic ALCL were retreated twice. Patients generally received brentuximab vedotin 1.8 mg/kg intravenously approximately every 3 weeks over 30 minutes as an outpatient infusion. The primary objectives of this study were to assess safety and to estimate antitumor activity of brentuximab vedotin retreatment.

**Results:**

The objective response rate was 60% (30% CR) in HL patients and 88% (63% CR) in systemic ALCL patients. The estimated median duration of response for patients with an objective response was 9.5 months (range, 0.0+ to 28.0+ months) at the time of study closure. Of the 19 patients with objective response, 7 patients had not had an event of disease progression or death at the time of study closure; duration of response for these patients ranged from 3.5 to 28 months. Of the 11 patients with CR, 45% had response durations of over 1 year.

Adverse events (AEs) occurring in ≥25% of patients during the retreatment period were generally similar in type and frequency to those observed in the pivotal trials of brentuximab vedotin monotherapy, with the exception of peripheral neuropathy, which is known to have a cumulative effect. Grade 3 or higher events were observed in 48% of patients; these were generally transient and managed by dose modifications or delays. Deaths due to AEs occurred in 3 HL patients; none were considered to be related to brentuximab vedotin retreatment.

**Discussion:**

With the exception of a higher rate of peripheral motor neuropathy, retreatment with brentuximab vedotin was associated with similar side effects seen in the pivotal trials.

**Conclusions:**

Retreatment with brentuximab vedotin monotherapy is associated with response rates in 68% (39% CR) of patients with relapsed HL and systemic ALCL.

**Trial registration:**

United States registry and results database ClinicalTrials.gov NCT00947856.

## Background

Brentuximab vedotin (ADCETRIS®) is an antibody-drug conjugate composed of a CD30-targeted chimeric monoclonal antibody (cAC10) covalently linked, via a protease-cleavable linker, to the microtubule-disrupting agent monomethyl auristatin E. Results from phase 2 pivotal trials of single-agent brentuximab vedotin (1.8 mg/kg) demonstrated an objective response rate (ORR) of 75% (34% complete remission [CR]) in Hodgkin lymphoma (HL) patients and 86% (57% CR) in systemic anaplastic large cell lymphoma (ALCL) patients [1,2]. The median duration of response for patients with an objective response was 6.7 months for HL patients and 12.6 months for systemic ALCL patients and the median duration of response for patients with CR was 20.5 months and 13.2 months, respectively [1,2]. Long-term follow-up date for these patients continue to show durable CR [3,4]. The safety profile was associated with manageable toxicities.

Physicians caring for HL and systemic ALCL patients who initially respond to brentuximab vedotin and then subsequently progress face a conundrum. Their choices range from aggressive regimens that enable transplantation to palliative measures with a goal of maximizing a patient’s quality of life. It was hypothesized that these patients might benefit from a second course of brentuximab vedotin.

This phase 2 study was designed to investigate the safety and antitumor activity of brentuximab vedotin when administered as a retreatment option for patients who had previously achieved an objective response (complete or partial remission [PR]) with prior brentuximab vedotin treatment. Secondary objectives were to assess the duration of tumor control, progression-free and overall survival, and the incidence of antitherapeutic antibodies (ATA).

## Methods

### Patient eligibility

Eligibility criteria for this study included patients who previously experienced a CR or PR with brentuximab vedotin, discontinued treatment while in remission, and subsequently experienced disease progression or relapse. Patients who received an allogeneic stem cell transplant (SCT) were eligible if they were >100 days from transplant and had no evidence of cytomegalovirus by polymerase chain reaction.

### Study design and treatment

This was an open-label, multicenter, international, phase 2 study of retreatment in patients who had responded to brentuximab vedotin monotherapy on a previous clinical trial. This report summarizes results of retreatment for patients with HL or systemic ALCL.

Patients in the retreatment arm were treated at 10 sites in the United States and 1 site in France. Patients began enrolling in July 2009. An interim analysis was performed on a subset of patients in the retreatment arm who had enrolled in the study and signed informed consent by April 2012. The Sponsor stopped the study in January 2013 because the pool of potential retreatment patients from prior trials was projected to be minimal. The final retreatment analysis was performed after study closure in March 2013. These data are presented in this article.

The protocol for this study was designed in accordance with the general ethical principles outlined in the Declaration of Helsinki. The conduct of all aspects of the study, including methods for obtaining informed consent, was also in accordance with principles enunciated in the declaration. The study was registered on ClinicalTrials.gov (NCT00947856) and the protocol was approved by the institutional review board or independent ethics committee for each study site; all patients provided written informed consent before any study-specific procedures began.

Retreatment with brentuximab vedotin monotherapy was administered intravenously over approximately 30 minutes on Day 1 of each 21-day treatment cycle (i.e., one dose is equal to one cycle), although investigators may have adjusted the dosing schedule as appropriate to manage side effects. Dosing was based on the patient’s weight, as measured according to institutional standards. The starting dose was to be 1.8 mg/kg; however, if for any reason a patient entered the study after receiving 1.2 mg/kg every 3 weeks on a prior trial, their dose continued to be 1.2 mg/kg. Patients were allowed to continue receiving treatment until disease progression, unacceptable toxicity, or study closure occurred; no maximum duration of therapy was specified in the protocol.

### Study assessments

The primary objective of the study was to evaluate the safety and antitumor activity of retreatment with brentuximab vedotin. The assessment of safety during the course of the study consisted of the surveillance and recording of adverse events (AEs), including any serious AEs (SAEs), recording of concomitant medication, and measurements of laboratory tests. A Safety Monitoring Committee assessed the safety and antitumor activity data periodically during the study.

Determination of antitumor activity was based on investigator assessment of response. The restaging schedule was performed per institutional standard of care; treatment decisions were based on the investigator assessment of response. Response was assessed using computed tomography (CT) and positron emission tomography (PET) according to the Revised Response Criteria for Malignant Lymphoma [5]. B symptoms (fever, night sweats, and weight loss) were also captured.

Plasma and serum samples for brentuximab vedotin concentration and immunogenicity evaluation were obtained prior to brentuximab vedotin administration in Cycles 1 and 2, and at the end of treatment visit.

Patients who discontinued treatment remained in study follow-up unless they withdrew consent and were contacted at least every 3 months until death or study closure. Patients who had a CR or PR in retreatment and then progressed after discontinuing therapy may have been re-enrolled and retreated more than once in this study at the discretion of the investigator.

Adverse events were classified using the Medical Dictionary for Regulatory Activities (MedDRA) version 14 and graded using the National Cancer Institute (NCI) Common Terminology Criteria for Adverse Events (CTCAE) Version 3.0; laboratory abnormalities were also graded using the NCI CTCAE. Concomitant medications were coded using World Health Organization (WHO) Drug (Version June 2009).

### Statistical analysis

Safety of retreatment with brentuximab vedotin was summarized descriptively for all patients by the type, incidence, severity, seriousness, and relatedness of AEs and laboratory abnormalities, including the incidence of ATA.

Antitumor activity was evaluated for patients with post-baseline response measurements by calculating the ORR, defined as the proportion of patients with CR or PR, and its two-sided 95% exact confidence interval (CI). Secondary objectives included the CR rate, duration of response, progression-free survival (PFS), and overall survival (OS). The duration of response, PFS, and OS were estimated using Kaplan-Meier methodology.

## Results

### Patients

Twenty-nine patients with HL (N = 21) or systemic ALCL (N = 8) were retreated in this study. Demographic and disease characteristics are presented in Table 1. The median age of HL and systemic ALCL patients was 33 years (range, 16 to 72 years). ECOG performance status was 0 or 1 in 93% of patients. Patients were a median of 4.3 years (range, <1 to 8.4 years) from time of disease diagnosis to retreatment. The median time from the last dose of brentuximab vedotin on the most recent study to the first dose of brentuximab vedotin on the retreatment study was 8 months (range, 2 to 45 months).

**Table 1:** Demographics and characteristics for retreatment baseline

**Characteristic**	**HL patients**	**ALCL patients**
	**(N = 21)**	**(N = 8)**
Age, year		
Median (range)	30 (16, 65)	51.5 (24, 72)
Gender, no. (%)		
Male	10 (48)	4 (50)
Female	11 (52)	4 (50)
Race, no. (%)		
Other	0 (0)	1 (13)
Black or African American	2 (10)	2 (25)
White	19 (90)	5 (63)
ECOG Performance Status, no. (%)		
0	8 (38)	3 (38)
1	12 (57)	4 (50)
2	1 (5)	1 (13)
Initial diagnosis, no. (%)		
ALK positive	--	3 (38)
ALK negative	--	5 (63)
Number of prior systemic therapies^a^		
Median (range)	4 (2, 12)	3 (2, 6)
Time between last brentuximab vedotin dose on prior study and first dose of retreatment (months)		
Median (range)	11.4 (4, 45)	4.7 (2, 15)
Number of patients with intervening systemic therapies, n (%)	6 (21)	0
Best response, n/n (%)		
Stable disease	1 (17)	--
Progressive disease	4 (67)	--
Unknown/other	1 (17)	--
Disease status relative to most recent prior therapy, n (%)		
Refractory	5 (24)	0
Relapse after response	16 (76)	8 (100)

^a^Includes any prior brentuximab vedotin treatment.

Patients had at least 2 prior systemic therapies (median, 4 systemic therapies; range, 2 to 12 systemic therapies), including at least one prior course of brentuximab vedotin therapy (Table 1). With the exception of 1 patient who had stable disease, all patients had a prior objective response to brentuximab vedotin. The patient had a best response of stable disease on the previous study. This patient had received 1.2 mg/kg every week for 3 weeks but missed at least 3 doses of study drug due an AE of neutropenia; the treating physician felt that the dosing schedule was suboptimal and an exemption was granted for the patient to enter the retreatment study. Six patients (21%) received intervening systemic therapies between the most recent prior brentuximab vedotin treatment and retreatment, including one patient who received commercial brentuximab vedotin. The best response achieved with the intervening systemic therapy was progressive disease (67%), stable disease (17%), or unknown/other (17%) for the 1 patient who had received commercial brentuximab vedotin treatment.

### Efficacy

**Figure 1:**
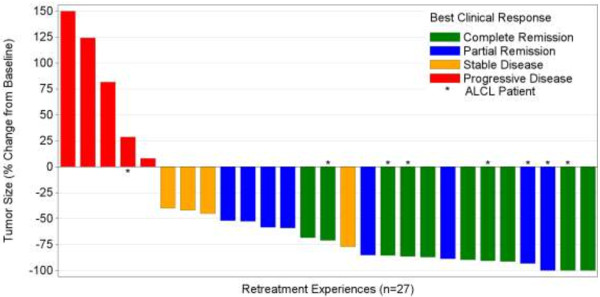
**Maximum reduction in target lesion size.** Best change in the sum of the product of diameter (SPD) of target lesions achieved over all restages. One patient with Hodgkin lymphoma had no postbaseline tumor measurements reported and is thus not included in the summary. Analysis excludes the second retreatment for 3 systemic anaplastic large cell lymphoma patients.

The efficacy evaluable population included 28 patients (20 HL and 8 systemic ALCL patients), as 1 HL patient did not have post-baseline response assessments. The ORR for HL and systemic ALCL retreatment patients was 68% (95% CI; 47.6, 84.1), with a CR rate of 39% (95% CI; 21.5, 59.4) (Table 2). The ORR was 60% (30% CR) for HL patients and 88% (63% CR) for systemic ALCL patients. The majority of patients (81%) who received brentuximab vedotin retreatment experienced reduction in measurable tumor volume (Figure 1).

**Table 2:** Key response results

**Parameter**	**HL patients**	**ALCL patients**
	**(N = 20)**	**(N = 8)**
Objective response rate (CR + PR)	12 (60)	7 (88)
Best clinical response^a^		
Complete remission	6 (30)	5 (63)
Partial remission	6 (30)	2 (25)
Stable disease	4 (20)	0
Progressive disease	4 (20)	1 (13)
95% CI for ORR^b^	36.1, 80.9	47.3, 99.7
95% CI for CR rate^b^	11.9, 54.3	24.5, 91.5
Duration of objective response for patients with OR, months^c^	12 (60)	7 (88)
Median (95% CI)^d^	9.2 (2.1, -)	12.3 (6.6, -)
Duration of response for patients with CR, months^c^	6 (30)	5 (63)
Median (95% CI)^d^	9.4 (1.7, 14.2)	12.9 (7.4, -)
Progression-free survival, months^e^		
Median (95% CI)^d^	9.9 (3.4, 13.4)	12.9 (1.4, 18.5)
Overall survival, months^e^		
Median (95% CI)^d^	- (11.4, -)	- (3.3, -)

Analysis excludes the second retreatment for 3 systemic anaplastic large cell lymphoma patients.

^a^Best response (according to Cheson 2007) prior to the start of any new antitumor treatment, exclusive of stem cell transplant.

^b^Two-sided 95% exact confidence interval (CI), computed using the Clopper-Pearson method (1934).

^c^Duration of response is calculated from the earliest occurrence of either complete or partial remission.

^d^Computed using the log-log transformation method of Collett (1994).

e As estimated using Kaplan-Meier methods.

**Figure 2:**
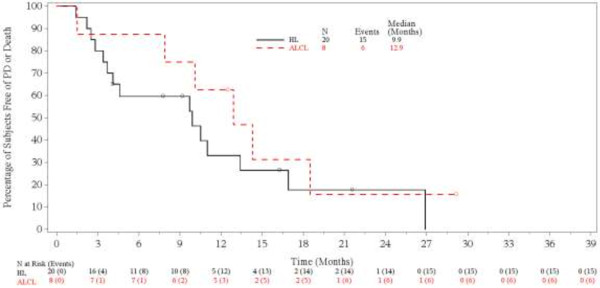
**Progression-free survival.** Symbols on the plot indicate censored patients. Analysis excludes the second retreatment for 3 systemic anaplastic large cell lymphoma patients.

**Figure 3:**
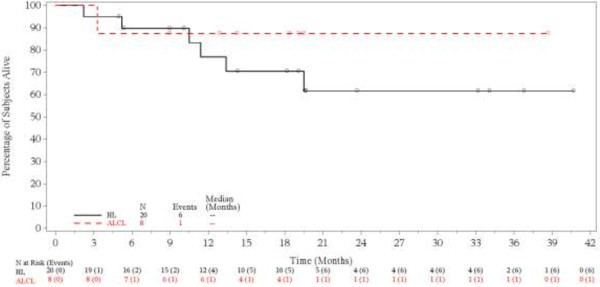
**Overall survival.** Symbols on the plot indicate censored patients. Analysis excludes the second retreatment for 3 systemic anaplastic large cell lymphoma patients.

The estimated median duration of response for patients with an objective response was 9.5 months (range, 0.0+ to 28.0+ months): this median duration was 9.2 months (range, 0.0+ to 19.5+ months) for HL patients and 12.3 months (range, 6.6 to 28.0+ months) for systemic ALCL patients. For patients with CR, the estimated median duration of response was 12.3 months (range, 7.4 to 14.2 months): this median duration was 9.4 months (range, 1.7 to 14.2 months) for HL patients and 12.9 months (range, 7.4 to 28.0+ months) for systemic ALCL patients. Twelve patients with objective response had an event of disease progression or death at the time of study closure, including 8 patients who had a best response of CR. The median PFS for HL and systemic ALCL patients was 9.9 and 12.9 months, respectively (Figure 2). The median OS had not yet been reached at the time of study closure and further long-term follow-up is not planned (Figure 3).

Ten patients had B symptoms at baseline. Resolution of all B symptoms occurred in 6 patients and occurred, on average, 5 weeks after initiation of retreatment with brentuximab vedotin. Of these 6 patients, 4 patients had an objective response and 2 patients had stable disease.

Two patients who were retreated with brentuximab vedotin achieved CR and subsequently received an allogeneic SCT. One patient with systemic ALCL had a CR after frontline treatment with brentuximab vedotin combined with CHOP and then relapsed; retreatment with brentuximab vedotin enabled a successful allogeneic SCT with a subsequent continuing duration of response greater than 1 year. One Stage IV HL patient with multiple prior therapies (doxorubicin, bleomycin, vinblastine, and dacarbazine; ifosfamide, carboplatin, and etoposide; gemcitabine) had a best response of PR with initial brentuximab vedotin treatment and then received an allogeneic SCT; this patient subsequently relapsed and was retreated with brentuximab vedotin. After achieving CR with retreatment, the patient had another allogeneic SCT with a duration of response of approximately 9 months prior to subsequent progression.

### Safety

Twenty-seven of the 29 patients started retreatment at 1.8 mg/kg and 2 patients started retreatment at 1.2 mg/kg (dose reduced in a prior study due to management of AEs). The median number of brentuximab vedotin cycles was 7 (range, 2 to 37 cycles). The median duration of retreatment was 5 months (range, 1 to 38 months).

Treatment-emergent AEs that occurred in ≥25% of patients were peripheral sensory neuropathy (59%); fatigue and nausea (41% each); diarrhea (38%); and arthralgia, headache, peripheral motor neuropathy, and pyrexia (28% each) (Table 3). Adverse events leading to retreatment discontinuation occurred in 9 patients (31%); 6 patients discontinued retreatment due to either peripheral sensory neuropathy (5 patients) or peripheral motor neuropathy (1 patient).

**Table 3:** Treatment-emergent adverse events reported by at least 20% of patients and grade 3 and higher incidence of these events

**Event term**	**Treatment-emergent adverse events (any grade)**	**Any grade 3 events**	**Any grade 4 events**	**Any grade 5 events**
Any event, n (%)	28 (97)	8 (28)	3 (10)	3 (10)
Peripheral sensory neuropathy	17 (59)	2 (7)	0	0
Fatigue	12 (41)	3 (10)	1 (3)	0
Nausea	12 (41)	1 (3)	0	0
Diarrhea	11 (38)	0	0	0
Arthralgia	8 (28)	2 (7)	0	0
Headache	8 (28)	0	0	0
Peripheral motor neuropathy	8 (28)	2 (7)	0	0
Pyrexia	8 (28)	0	0	0
Anemia	7 (24)	5 (17)	0	0
Dyspnea	7 (24)	1 (3)	1 (3)	0
Back pain	6 (21)	1 (3)	0	0

Events that were ≥ Grade 3 occurred in 48% of patients and event terms occurring in ≥10% of patients were anemia (17%); fatigue (14%); and hyperglycemia, hypophosphatemia, and thrombocytopenia (10% each). Serious AEs were defined as AEs that were fatal, life threatening, disabling/incapacitating, medically significant, or led to hospitalization or a congenital anomaly or birth defect and occurred in 8 patients. Of these, Grade 5 (fatal) events occurred in 3 HL patients and were due to respiratory failure, graft versus host disease, or bronchopulmonary aspergillosis; none of these fatal events were considered to be related to brentuximab vedotin retreatment. Additionally, 6 patients died after the 30-day safety follow-up period; 4 patients due to disease progression, 1 patient who experienced sepsis, and 1 patient with an unknown etiology.

Almost half the patients who entered the study had pre-existing peripheral neuropathy (48%) at retreatment baseline. Treatment-emergent peripheral neuropathy events were defined as newly occurring (not present at retreatment baseline) or worsening after the first dose of retreatment. Peripheral neuropathy was observed in 69% of patients and included peripheral sensory neuropathy, peripheral motor neuropathy, paraesthesia, burning sensation, and gait disturbance; severity was generally Grade 1 or 2. Grade 3 peripheral neuropathy events occurred in 3 patients; the median time to onset of Grade 3 events was approximately 13 months (range, 6 to 20 months). Peripheral neuropathy events were managed with dose modifications (delays or reductions).

Of the 28 patients with any immunogenicity results, 12 patients (43%) tested positive for ATA at any visit during the study and 9 patients (32%) tested positive for ATA at baseline. Six HL patients had infusion-related reactions; of these, 5 patients tested positive for ATA post-baseline. Of the 11 patients with a best response of CR, 7 patients tested positive for ATA at least once during the study. None of the 5 patients with a best response of progressive disease tested positive for ATA at any visit.

### Patients retreated more than once

Three systemic ALCL patients were retreated twice during the study; all 3 patients were female and were 52, 53, and 70 years of age. Safety profiles were generally similar for both the first and second retreatment experiences. Two patients achieved a PR with both retreatment experiences and 1 patient achieved a CR with both retreatment experiences. None of these patients developed ATA during either retreatment experience.

## Discussion

This open-label multicenter study evaluated the safety and antitumor activity of retreatment with brentuximab vedotin in patients with relapsed HL or systemic ALCL who had responded previously to brentuximab vedotin therapy. Antitumor activity was observed in 68% of HL and systemic ALCL retreatment patients. At the time of study closure, the estimated median duration of response for patients with an objective response was 9.5 months. Seven of 19 patients with an objective response were still alive and in remission at the time of study closure; duration of response for these patients ranged from 3.5 to 28 months. The antitumor activity observed with retreatment is consistent with the objective response rates seen in the phase 2 pivotal trials in patients with relapsed or refractory HL (75%) and systemic ALCL (86%). In the pivotal trials, the median duration of objective response for patients with CR was 20.5 months for HL patients and 13.2 months for systemic ALCL patients [1,2]; long-term follow up data continue to show durable CR [3,4]. Unlike the pivotal trials, patients in the retreatment study were scanned at intervals per institutional standard of care; thus, remission durability is less precise.

Upon retreatment, observed AEs (occurring in ≥25% of patients), including peripheral sensory neuropathy, fatigue, nausea, diarrhea, arthralgia, headache, peripheral motor neuropathy, and pyrexia, were typically managed with dose modifications and were primarily Grade 1 or 2 in severity. The rates for AEs reported in patients retreated with brentuximab vedotin were generally consistent with those observed in the pivotal phase 2 trials, with the exception of peripheral motor neuropathy seen in a higher percentage of patients (28% total versus 11% for HL patients and 5% for systemic ALCL patients in the pivotal trials); these events were primarily Grade 1 or 2 in severity [1,2].

There is precedent in lymphoma for retreatment with an effective targeted agent as retreatment data with rituximab in patients with relapsed low-grade or follicular, CD20-positive B-cell non-Hodgkin lymphoma resulted in an ORR of 38% for retreatment [6]. In the current study, 60% of patients with HL and 88% of patients with systemic ALCL responded to retreatment with brentuximab vedotin, often despite a short remission duration (less than 3 months) following initial brentuximab vedotin treatment.

Whether allogeneic transplantation should be employed after a failed autograft is a subject of considerable uncertainty. In this present study, complete remissions with a median duration of over 1 year were obtained by 45% of retreatment patients, 2 of whom subsequently received allogeneic SCT. Similarly, few allografts were performed in the 102 patient relapsed/refractory HL pivotal trial that led to the initial approval of brentuximab vedotin and this approach appeared to be associated with acceptable long-term outcomes. In a 3-year follow-up analysis, 50% of patients remained alive and 18 of all 102 treated patients remained in remission per investigator review [3]. In a separate case review that evaluated brentuximab vedotin in heavily-pretreated (5–19 prior therapies) HL patients with recurrent disease after allogeneic SCT, 50% of patients achieved an objective response (38% CR) [7]. However, 50% to 60% of patients who undergo allogeneic SCT have chronic graft versus host disease, which often represents a significant burden on their quality of life [8]. Another possibility could be retreatment with brentuximab vedotin and consideration of consolidation with allogeneic transplant at the time of the next remission [9]. Preliminary results from a retrospective analysis of relapsed or refractory HL patients suggest that, after 2 years of follow-up, brentuximab vedotin prior to reduced intensity allogeneic hematopoietic cell transplantation may yield prolonged disease control without a delay in engraftment, increases in non-relapse mortality, or other post-transplant complications [10]. One consideration during retreatment is that patients should be followed closely for peripheral sensory or motor neuropathy and dose reductions initiated as needed.

The findings from this study suggest that brentuximab vedotin retreatment is a viable option for a relatively young, heavily pretreated lymphoma patient population who presently struggle with the toxic effects of serial, palliative chemotherapeutic regimens in an attempt to maximize quality of life.

## Conclusions

In summary, retreatment with brentuximab vedotin delivered a second response in 68% (39% CR) of HL and systemic ALCL patients who had previously received treatment. With the exception of a higher rate of peripheral motor neuropathy, retreatment with brentuximab vedotin was associated with similar side effects seen in the pivotal trials.

## Abbreviations

AE: Adverse event; ALCL: Anaplastic large cell lymphoma; ATA: Antitherapeutic antibody; CI: Confidence interval; CR: Complete remission; CT: Computed tomography; CTCAE: Common terminology criteria for adverse events; ECOG: Eastern cooperative oncology group; EHA: European hematology association; HL: Hodgkin lymphoma; MedDRA: Medical dictionary for regulatory activities; NCI: National cancer institute; ORR: Objective response rate; OS: Overall survival; PET: Positron-emission tomography; PFS: Progression-free survival; PR: Partial remission; SAE: Serious adverse event; SCT: Stem cell transplant; WHO: World health organization.

## Competing interests

NLB has received research funding/grants, travel expenses, and consultancy fees from Seattle Genetics, Inc. RC has received research funding/grants, travel expenses, and consultancy and speaker’s bureau fees from Seattle Genetics, Inc. MAF has received research funding/grants, travel expenses, consultancy fees, and honoraria, from Seattle Genetics, Inc. and has acted in an advisory capacity for Seattle Genetics Inc. PB and JVM have received research funding/grants and honoraria from Seattle Genetics, Inc. AG has received research funding/grants from Seattle Genetics, Inc., Cephalon, Piramal, Merck, Calistoga, Pfyzer, Abbott, and Spectrum; speaker’s bureau fees from Takeda Pharmaceuticals International Co., Amgen, Genzyme, and Cellular Therapeutics, Inc.; honoraria from Seattle Genetics, Inc. and Takeda Pharmaceuticals International Co., and consultancy fees from Seattle Genetics, Inc. SES has received research funding/grants from Seattle Genetics, Inc.; speaker’s bureau fees from Celgene, GlaxoSmith Kline, and Cephalon; and consultancy fees from Celgene, Spectrum, and Cephalone. RA has received research funding/grants and consultancy fees from Seattle Genetics, Inc. and Takeda Pharmaceuticals International Co., and has acted in an advisory capacity for Seattle Genetics, Inc. JR and AFT have received research funding/grants from Seattle Genetics, Inc. RR has received research funding/grants and speaker’s bureau fees from Seattle Genetics, Inc. DH is employed by and has equity ownership (including stock options) in Takeda Pharmaceuticals International Co. PL and LG are employed by and have equity ownership (including stock options) in Seattle Genetics, Inc.

## Authors’ contributions

NLB, RC, MAF, PB, AG, SES, RA, JVM, RR, JR, and AFT contributed to the acquisition of the data. PL contributed to the analysis and interpretation of the data. All authors contributed to the concept and design of the study, the analysis and interpretation of the data, and manuscript drafts and revisions. The final manuscript was read and approved by all authors.

## Research support

Direct funding for this research was issued by Seattle Genetics, Inc. and supported by the joint financial resources of Seattle Genetics, Inc. and Takeda Pharmaceuticals International Co.

## Related presentations

Presented in part at the annual ASCO (2010 and 2012), ISHL (2010), and EHA (2012) meetings.
